# Exploring the relationship between general practice characteristics and attendance at Walk-in Centres, Minor Injuries Units and Emergency Departments in England 2009/10–2012/2013: a longitudinal study

**DOI:** 10.1186/s12913-017-2483-x

**Published:** 2017-08-08

**Authors:** Peter Tammes, Richard W. Morris, Emer Brangan, Kath Checkland, Helen England, Alyson Huntley, Daniel Lasserson, Fiona MacKichan, Chris Salisbury, Lesley Wye, Sarah Purdy

**Affiliations:** 1 Centre for Academic Primary Care, Bristol Medical School, University of Bristol, Bristol, UK; 2 Centre for Primary Care, Institute of Population Health, University of Manchester, Manchester, UK; 3 Devon Partnership NHS Trust, Exeter, UK; 4 Nuffield Department of Medicine, University of Oxford, Oxford, UK

**Keywords:** Longitudinal study, ‘Emergency service, hospital’, Primary health care, Population characteristics, Multilevel modelling, Continuity of care, Alternative health care service

## Abstract

**Background:**

The UK National Health Service Emergency Departments (ED) have recently faced increasing attendance rates. This study investigated associations of general practice and practice population characteristics with emergency care service attendance rates.

**Methods:**

A longitudinal design with practice-level measures of access and continuity of care, patient population demographics and use of emergency care for the financial years 2009/10 to 2012/13. The main outcome measures were self-referred discharged ED attendance rate, and combined self-referred discharged ED, self-referred Walk-in Centre (WiC) and self-referred Minor Injuries Unit (MIU) attendance rate per 1000 patients. Multilevel models estimated adjusted regression coefficients for relationships between patients’ emergency attendance rates and patients’ reported satisfaction with opening hours and waiting time at the practice, proportion of patients having a preferred GP, and use of WiC and MIU, both between practices, and within practices over time.

**Results:**

Practice characteristics associated with higher ED attendance rates included lower percentage of patients satisfied with waiting time (0.22 per 1% decrease, 95%CI 0.02 to 0.43) and lower percentage having a preferred GP (0.12 per 1% decrease, 95%CI 0.02 to 0.21). Population influences on higher attendance included more elderly, more female and more unemployed patients, and lower male life-expectancy and urban conurbation location. Net reductions in ED attendance were only seen for practices whose WiC or MIU attendance was high, above the 60th centile for MIU and above the 75th centile for WiC. Combined emergency care attendance fell over time if more patients within a practice were satisfied with opening hours (−0.26 per 1% increase, 95%CI −0.45 to −0.08).

**Conclusion:**

Practices with more patients satisfied with waiting time, having a preferred GP, and using MIU and WIC services, had lower ED attendance. Increases over time in attendance at MIUs, and patient satisfaction with opening hours was associated with reductions in service use.

**Electronic supplementary material:**

The online version of this article (doi:10.1186/s12913-017-2483-x) contains supplementary material, which is available to authorized users.

## Background

In many developed countries emergency department (ED) attendance is increasing [1]. The increase in the number of attendees at EDs in England over recent years has resulted in considerable pressure on these services,^1^ and is a major concern for National Health Service (NHS) sustainability. A considerable number of the attendees at EDs are self-referred patients, and various recently conducted research showed that the percentage of patients attending EDs that could be managed in English general practices ranged from 15% to 66% [2–4]. We have followed up on these findings, by investigating associations between general practice-related factors and ED attendance over time in order to highlight potential measures to reduce the pressure on ED.

A recent systematic review identified general practice factors which affect ED attendance [5]. The limitations of this review were that the studies selected were mainly cross-sectional with some only reporting univariable associations, while others focused on specific geographic areas or patient populations by condition. A more recent cross-sectional study partially replicated the findings from this systematic review but importantly included previously untested factors in a contemporary England wide context [6]. General practice factors which affected ED attendance were access related features such as being able to make a convenient appointment and to see a nurse/GP at short notice, continuity of care such as being able to see a preferred GP often when visiting the practice, and composition of GP staff in the practice such as proportion of UK-qualified GPs. Factors related to the general practice population such as usage of alternative health care services, i.e. Walk-in Centres (WiC) or Minor Injuries Units (MIU), and socio-demographics such as ethnicity, unemployment, and life-expectancy were also related to practices’ ED attendance rates. Furthermore, general practices located in urban conurbation areas showed higher ED attendance rates.

By conducting a longitudinal study, at a practice level with repeated measures for the financial years 2009/10 to 2012/13, we are able to draw stronger inference from the calculated associations than from any previous cross-sectional study [7]. Also we have a nation-wide focus and include multiple general practice and practice population characteristics. The study’s aim is to investigate the impact of general practice and population characteristics on ED attendances in a longitudinal design, particularly to investigate the impact of changes within general practices and to test previous findings of cross-sectional studies in a nation-wide context over several years.

## Methods

### Study design and setting

This study used a longitudinal design with general practices in England as the unit of analysis for the time-period 2009/10 to 2012/13. For each financial year, practices were included when they were operative over the whole year, had more than 500 patients and had no missing values on the characteristics to be included in the models.^2^ Furthermore, practices were only included if their patients’ response rate to the GP Patient Survey (GPPS) was 20% or higher with a minimum of 100 completed survey forms. The GPPS is an independent survey carried out on behalf of NHS England, send out to over a million people every year across the UK since 2007, with the aim of determining how people feel about their general practice. Moreover, practices were only included if they had a standardized self-referred discharged ED rate above 10 per 1000 patients, since lower attendance rates would be implausible and probably occurred because of issues relating to recording or coding. As a result of these exclusion criteria the number of practices vary over those 4 years with a minimum of 5711 (71.6% of all operative general practices) in 2009/10 and a maximum of 7091 practices (87.9% of all operative practices) in 2012/13 (Table 1). All data were abstracted from publicly-available websites and comprised aggregated general practice-level data where patients could not be identified.

**Table 1:** Descriptive statistics general practices in England, longitudinal study 2009/10–2012/13

	2009/10 (*N* = 5711)		2010/11 (*N* = 6793)		2011/12(*N* = 6903)		2012/13 (*N* = 7091)	
	Median	Interquartile range	Median	Interquartile range	Median	Interquartile range	Median	Interquartile range
General practice population	6807	3650, 9153	6340	3822, 9582	6439	3925, 9717	6459	3929, 9736
Response rate GPPS	42.7	34.3, 49.1	40.9	32.9, 47.5	42.1	35.2, 48.5	39.6	32.1, 46.0
Pct. of patients 65+	34.1	28.5, 38.3	34.3	29.3, 38.4	34.6	29.9, 38.5	34.5	29.7, 38.5
Pct. of patients female	57.4	55.6, 59.1	57.5	55.9, 59.1	57.6	56.0, 59.1	57.5	55.9, 59.1
Pct. of patients UK white	91.5	74.5, 95.5	92.1	79.6, 95.6	92.6	82.6, 95.7	92.5	81.9, 95.7
Pct. of patients unemployed	3.5	2.2, 6.1	3.3	2.1, 5.6	3.2	2.0, 5.2	3.2	2.0, 5.3
Male life expectancy 2006–2010	78.1	76.3, 79.8	78.2	76.4, 79.8	78.3	76.5, 79.8	78.3	76.5, 79.8
Pct. of patients satisfied with open. Hours	82.9	78.9, 86.4	83.0	79.3, 86.5	83.3	79.5, 86.9	83.2	79.4, 86.8
Pct. of patients felt wait far too long	5.1	2.9, 8.6	5.1	2.9, 8.4	4.9	2.7, 8.1	4.9	2.8, 8.1
Pct. of patients having pref. GP	62.0	54.2, 68.7	62.6	54.6, 69.1	62.8	54.9, 69.3	62.8	54.8, 69.3
Pct. of patients saw pref. GP often	73.2	62.8, 82.5	73.5	63.1, 82.6	74.0	63.9, 83.1	73.9	63.8, 83.1
Std. self-ref. WiC attendance rate	0.1	0.0, 0.3	0.1	0.0, 0.3	0.1	0.0, 0.3	0.1	0.0, 0.3
Std. self-ref. MIU attendance rate	3.2	1.3, 23.5	3.0	1.3, 21.9	3.0	1.3, 22.3	3.0	1.3, 21.6
Std. self-ref. discharged ED attendance rate per 1000 general practice population	81.2	45.9, 116.0	85.8	56.2, 120.1	90.3	64.1, 124.9	92.3	63.0, 126.3
Combined ED, MIU & WiC attendance rate per 1000 general practice population	93.6	57.8, 130.4	99.9	69.5, 136.7	116.1	84.3, 154.6	123.5	89.9, 166
Rurality	Pct.		Pct.		Pct.		Pct.	
Urban conurbation	50.2		44.7		42.9		43.0	
cities and towns	35.9		40.4		40.9		41.3	
rural	13.9		14.9		16.1		15.7	

### Outcome measures

1. Self-referred discharged ED attendances per 1000 general practice population standardized according to age and gender at ‘major’ (type 1; see Additional file 1: Box 1) accident and emergency (A&E) departments either with or without practice follow-up treatment. These visits were identified as likely to be suitable for treatment by another health care service such as a GP, MIU or WiC [3]. ED attendances resulting in admission, onward referral, transfer to another provider, or death were excluded from this measure.
2. Combined ED, WiC and MIU attendance rate per 1000 general practice population standardized according to age and gender. WiC and MIU attendances resulting from referrals from emergency services, GPs, or other or unknown sources were excluded. Whereas consultant led single specialty accident and emergency services are classified as type 2 A&E departments, MIU and WiC are generally classified as types 3 and 4 A&E departments (see Additional file 1: Box 1). This combined outcome explores the association between general practice factors and a wider range of emergency care provision.

ED attendance rate, and WiC and MIU attendance rates per 1000 patients at general practice level were obtained from NHS Comparators.^3^

### Measures of English general practice characteristics

The main source providing GP characteristics and general practice population characteristics over time in this study is the GPPS.^4^ The GPPS response rates were quite stable within individual general practices over the four financial years in our analysis (Table 1). However, several survey questions changed over our time-period limiting the choice of characteristics to be included in our longitudinal analysis.^5^ Furthermore, we used the presented GPPS unweighted survey results as a result of a change in the GPPS weighting scheme from survey 2011/12 onwards resulting in incomparability of the data over time, even in cases where the same questions have been asked.^6^ Characteristics of interest in this study were mainly those where responses might change during our time-period, i.e. patients’ satisfaction with opening hours, percentage of patients having a preferred GP, unemployment rates due to the economic crisis, and percentage of patients being 65 or older due to the post-WWII baby boom generation.

#### Access to English general practice

This study used patients’ opinion on waiting times in the waiting room of general practices when having a booked-appointment, and patients’ satisfaction with opening hours.^7^ Furthermore, it used urban and rural English postcodes from the Office of National Statistics linked to GP postcodes to identify general practice locations.^8^

#### Continuity of care

To determine the degree of continuity of care this study used the percentage of patients having a preferred GP and of those, the percentage that could see or speak to their preferred GP always or a lot of the time.^9^

### Measures of English general practice population demographics

The index of multiple deprivation (IMD) is commonly used to characterize socio-demographic profiles. However, IMD includes standardized emergency admission rates as part of the health factor in its definition.^10^ In this study, life-expectancy was used as a health indicator as it is often used to show inequalities in health within countries, and is a more direct measure of health need in a population than the IMD. Data were used from Public Health England to determine the male life-expectancy among practice populations,^11^ which correlates more strongly with self-referred ED attendance rates than female life-expectancy. Unemployment rate was used as an indicator for economic status. Unemployment rates, the percentage of patients 65 years of age or older, and the percentage of UK-whites (that is respondents who identified themselves with White-English, Welsh, Scottish, Northern Irish, or British) among practice populations were obtained from GPPS.^12^

### Measures of local availability of MIU and WiC and their attendance rates

Self-referred attendance per 1000 general practice population data standardized according to age and gender for both MIU and WiC were obtained from the NHS Comparators website.^13^ When defining the presence of a WiC near a general practice, this was indicated by a WiC attendance of greater than one per 1000, otherwise a nearby WiC was assumed to be absent. We used an analogous definition for the presence of a MIU.

### Statistical methods

A multilevel model was used to analyze repeated measurements over time [8]. One of the advantages is that it can use data for general practices with incomplete data for all 4 years. Therefore we conducted multilevel analyses to examine the association of changes in the described characteristics over time with difference in emergency care use, whereby time (i.e. years) was the level 1 unit, general practices were the level 2 unit, and Primary Care Trusts (PCT) were the level 3 unit.^14^ All analyses were undertaken in Stata 13 MP2 (StataCorp, College Station, Texas, USA). For each predictor we included the time-specific value for each general practice and the time-average value. For example, for the percentage of female patients, the time specific value refers to the percentage in each financial year at that practice, while the time average value refers to the average percentage over all four financial years. We were most interested in the effect of the time-specific indicators on emergency care use, since these reflect effects of changes in the indicator on changes in ED attendance. Time-average effects relate mainly to effects of the average level of the indicator over the four years to average ED attendance over the four years, akin to an aggregated cross-sectional analysis. Adjusted regression coefficients, confidence intervals and exact *p*-values were tabulated for each predictor.

## Results

### Time-trend

**Fig. 1:**
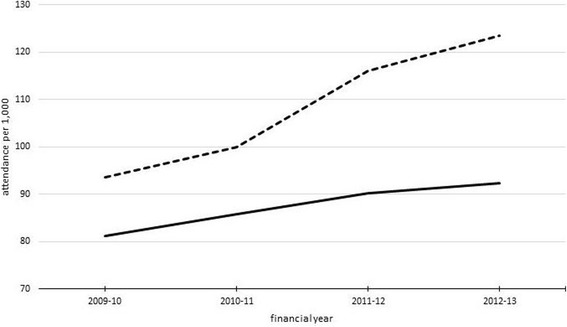
Trend in ED attendance rate and the combined ED, MIU & WiC attendance rate

Figure 1 shows the increase in ED and the combined ED, MIU & WiC attendance rates based on the rates presented in Table 1. The ED attendance rate increased between the financial years 2009/10 and 2011/12 where after the attendance rate became more stable. For the combined ED, MIU & WiC variable, however, a further increase occurred after 2011/12.

### Variances in English general practice and population characteristics

In Table 2 we present the between and within general practice variances of practice and population characteristics. Most variables show greater variance between practices than within practices. However, MIU attendance rate and WiC attendance rate showed substantial higher variance within practices than between practices. These last factors therefore carry most potential for investigating effects on changes within practices on ED attendance over time.

**Table 2:** Multilevel model including only the four financial years to determine the between and within general practice variances of practice and population characteristics

*N* = 26,498	Between PCT variance	Between general Practice variance	Within general practice variance
Time specific pct. 65+	25.7	37.7	7.6
Time specific pct. Female patients	0.8	5.9	8.3
Time specific pct. UK-white patients	327.7	126.4	7.0
Time specific pct. Unemployed patients	4.7	5.2	2.6
Time specific pct. Satisfied open hours	6.5	25.7	11.2
Time specific pct. Waited too long	4.8	23.1	5.3
Time specific pct. Had pref. GP	16.6	147.6	21.0
Time specific pct. Often spoke to pref. GP	19.3	170.8	33.8
Time specific WiC attendance rate	184.4	0	441.1
Time specific MIU attendance rate	902.7	872.9	1006.6

### Self-referred discharged ED attendance

#### Between practice variance: Time average effects of the general practice characteristics

Characteristics associated with lower self-referred discharged ED attendance rates included practices with lower percentage of elderly, lower percentage of female patients, lower patient population’s unemployment rates, lower percentage of patients dissatisfied with waiting time in waiting room, higher percentage of patients having a preferred GP, higher patient population’s MIU and WiC attendance rates, higher male patient population’s life expectancy, and general practices located outside cities and towns (model S1 in Additional file 1: Table S2). When including time specific effects of those indicators, the significance level of those time average effects did not change (see model S2 in Additional file 1: Table S2). However, the effects of dissatisfaction with waiting time in waiting room and the MIU attendance rate had less impact.

Including local availability of MIU or WiC did not alter the results significantly as presented in Table 3. Although, overall, general practices where more patients attended MIUs or WiCs tended to have lower rates of self-referred discharged ED attendance, conversely the presence of a MIU or WiC close to the practice was associated with an increase in ED attendance. The GPPS response rate was inversely associated with self-referred discharged ED attendance rates (model S3 in Additional file 1: Table S2). In this model dissatisfaction with waiting time in waiting room and having a preferred GP were less strongly associated with attendance rates.

**Table 3:** Estimates of B-coefficients from multilevel regression models for the association between general practice characteristics and socio-demographic profile of patients and ED attendance in England, 2009/10–2012/13

Fixed part	b-coef.	95% CI
Intercept	432.194***	
2010–11 ref. 2009–10	9.624***	8.672,10.575
2011–12 ref. 2009–10	17.340***	16.120,18.560
2012–13 ref. 2009–10	18.279***	16.843,19.714
Time specific pct. 65+	−0.044	−0.176,0.089
Time average pct. 65+	0.677***	0.502,0.852
Time specific pct. Female patients	−0.061	−0.184,0.063
Time average pct. Female patients	0.393**	0.124,0.662
Time specific pct. UK-white patients	−0.063	−0.198,0.072
Time average pct. UK-white patients	−0.008	−0.161,0.146
Time specific pct. Unemployed patients	−0.011	−0.233,0.211
Time average pct. Unemployed patients	2.255***	1.822,2.688
Time specific pct. Satisfied open hours	−0.092	−0.204,0.019
Time average pct. Satisfied open hours	0.007	−0.163,0.177
Time specific pct. Waited too long	0.099	−0.061,0.258
Time average pct. Waited too long	0.224*	0.015,0.433
Time specific pct. Had pref. GP	0.013	−0.065,0.091
Time average pct. Had pref. GP	−0.116*	−0.209,-0.024
Time specific pct. Often spoke to pref. GP	−0.047	−0.110,0.017
Time average pct. Often spoke to pref. GP	0.036	−0.047,0.118
Time specific WiC attendance rate	−0.011	−0.028,0.007
Time average WiC attendance rate	−0.219***	−0.279,-0.160
Time specific MIU attendance rate	−0.205***	−0.216,-0.194
Time average MIU attendance rate	−0.063***	−0.084,-0.041
Cities and towns ref. urban conurbation	4.083**	1.237,6.930
Rural areas ref. urban conurbation	−8.406***	−11.559,-5.254
Male life expectancy 2006/10	−4.860***	−5.291,-4.428
local availability of MIU	2.108***	1.082,3.134
local availability of WiC	4.025***	2.518,5.532
Random components of variance		
*PCT level:* Intercept	862.313***	664.770,1059.857
*General practice level:* Intercept	465.105***	443.814,486.395
*Year level:* Intercept	629.433***	616.768,642.099
Statistics		
N	26,498	
deviance	256,143.23	
Log likelihood	−128,071.62	

*** *p* < 0.001, ** *p* < 0.01, * *p* < 0.05

#### Within practice variance: time specific effects of the general practice characteristics

When patients within a practice show an increase in MIU attendance rate over the years then self-referred discharged ED attendances rate changed by −0.21 (95%CI -0.22 to −0.19) per extra MIU attendance (model S2 in Additional file 1: Table S2): this implied that for every extra 5 MIU attendances, one fewer ED attendance would occur. Estimates of time specific effects barely altered when GPPS response rates were included (see model S3 in Additional file 1: Table S2). We further investigated whether the time specific MIU attendance rate differed according to urban classification by adding an interaction term to our model. The same increase in MIU attendance rate was associated with a change in self-referred discharged ED attendance of −0.16 in urban conurbations, of −0.249 for practices in urban areas, and of −0.22 for practices in rural areas (*p*-value for interaction <0.0001); increased MIU attendance had greater impact outside urban conurbations.

Using the results shown in Table 3, we determined the relationship between predicted reduction in self–referred discharged ED attendance rate in a general practice population and activity at MIUs and WiCs in the general practice area (Additional file 1: Figure S1). Reductions in self-referred discharged ED attendance rate only began to be seen when the time specific MIU attendance was beyond 6.7 per 1000 patients, a situation only true for 40% of practices, and when time specific WiC attendance was greater than 16 per 1000, a situation true for only 25% of practices (see Additional file 1: Table S1). A greater effect on reductions in ED attendance were seen for MIU attendance than for WiC attendance. To elaborate further on the impact of availability and use of alternative care services, we compared the trend in self-referred discharged ED attendance rate for practices which never had a MIU and WiC around with practice that acquired a MIU or a WiC after 2009/10. General practices which acquired a MIU after 2009/10 showed a lower and parallel trend in ED attendance while practices which acquire a WiC after 2009/10 showed a steeper increase in self-referred discharged ED attendance rates after 2011/12 (Additional file 1: Figure S2).

### Combined self-referred discharged ED and self-referred WiC and MIU attendances

*Between practice variance*: time average effects of the general practice characteristics.

Characteristics associated with lower emergency care attendance rates included practices with lower percentage of elderly, lower percentage of female patients, lower patient population’s unemployment rates, lower percentage of patients dissatisfied with waiting time in waiting room, higher male patient population’s life expectancy, and general practices located outside urban conurbation areas (model S1 in Additional file 1: Table S3). When including time specific effects of those indicators (Table 4), a lower proportion of UK white patients was associated with lower emergency care attendance rates but results for other indicators did not change.

**Table 4:** Estimates of B-coefficients from multilevel regression models for the association between general practice characteristics and socio-demographic profile of patients and combined ED, MIU & WiC attendance in England, 2009/10–2012/13

Fixed part	b-coef.	95% CI
Intercept	461.627***	
2010–11 ref. 2009–10	11.973***	10.445,13.502
2011–12 ref. 2009–10	33.744***	31.894,35.594
2012–13 ref. 2009–10	43.880***	41.722,46.038
Time specific pct. 65+	−0.029	−0.244,0.186
Time average pct. 65+	1.071***	0.801,1.342
Time specific pct. Female patients	−0.087	−0.288,0.114
Time average pct. Female patients	0.530**	0.133,0.927
Time specific pct. UK-white patients	−0.338**	−0.558,-0.119
Time average pct. UK-white patients	0.366**	0.123,0.609
Time specific pct. Unemployed patients	−0.094	−0.455,0.267
Time average pct. Unemployed patients	3.153***	2.507,3.798
Time specific pct. Satisfied open hours	−0.264**	−0.445,-0.082
Time average pct. Satisfied open hours	0.107	−0.151,0.365
Time specific pct. Waited too long	−0.034	−0.294,0.226
Time average pct. Waited too long	0.542**	0.218,0.866
Time specific pct. Had pref. GP	0.050	−0.077,0.176
Time average pct. Had pref. GP	−0.113	−0.258,0.033
Time specific pct. Often spoke to pref. GP	0.022	−0.081,0.125
Time average pct. Often spoke to pref. GP	−0.030	−0.158,0.098
Cities and towns ref. urban conurbation	−5.350**	−9.361,-1.339
Rural areas ref. urban conurbation	−18.288***	−22.740,-13.836
Male life expectancy 2006/10	−5.446***	−6.062,-4.830
Random components of variance		
*PCT level:* Intercept	1590.901***	1225.077,1956.726
*General practice level:* Intercept	833.953***	790.224,877.681
*Year level*: Intercept	1675.340***	1641.657,1709.024
Statistics		
N	26,498	
deviance	280,065.22	
Log likelihood	−140,032.61	

*** *p* < 0.001, ** *p* < 0.01

#### Within practice variance: Time specific effects of the general practice characteristics

Changes in the proportion of UK white patients and satisfaction with opening hours within a practice are associated with changes in emergency care attendance rates (Table 4). When practices show an increase in the proportion of UK white patients over the years then emergency care service attendance rates falls. When more patients within a practice are satisfied with opening hours over the years then emergency care attendance rates fall. The GPPS response rate was inversely associated with emergency care attendance rates but this did not affect estimates of the impact of other variables (model S2 in Additional file 1: Table S3).

## Discussion

The results show that differences between general practices in their patients’ attendance rates at urgent care services including ED, MIU and WIC between 2009/10 and 2012/13 are related to elements of general practice provision as well as socio-demographic characteristics of the practice population and geographical factors. General practice characteristics associated with higher ED attendance rates included a lower percentage of patients satisfied with waiting time in the waiting room, and a lower percentage of patients having a preferred GP. General practice population characteristics including higher unemployment rate, a higher percentage of elderly patients, a higher percentage of female patients and lower male life-expectancy were also associated with higher ED attendance rates.

Furthermore, general practices where more patients attended MIUs and WiCs were associated with lower emergency attendance, but only for those practices whose attendance at such services was high. General practices whose MIU attendance increased over time saw a further decrease in ED attendance, especially those located outside urban conurbations. Combined emergency care attendance fell over time if more patients within a general practice were satisfied with the practice opening hours. Those services were also less frequently visited when the proportion of UK-white patients within a general practice increased over time.

### Strengths and limitations

To our knowledge, this is the first longitudinal study involving repeated measures of emergency care use together with potential explanatory variables. Many cross-sectional analyses have been carried out, [3, 9–11] but very few have investigated more than 1 year of data. Some studies have presented a series of year-on-year emergency care use data, looking particularly for changes associated with a discrete policy change [12–16]. The present study goes beyond cross-sectional associations which are especially prone to confounding and highlights changes in emergency care use associated with changes in key general practice characteristics.

Data on key indicators were merged from different sources in one database and thus the information used included both survey and routine data. The GPPS median response rates varied over the years between 39.6% and 42.7%. Response rates showed an inverse correlation with self-referred discharged ED attendance rates. Including response rates as a covariate in the models led to a reduced estimate of the effect of having a preferred GP and waiting time in waiting room at the general practice. This might indicate that response rate confounds the relationship between waiting time and ED attendance and especially the relationship between having a preferred GP and ED attendance. General Practices with higher GPPS response rate might indicate more committed or attending patients resulting in both more patients having a preferred GP and lower general practices’ ED attendance rates. General practices with higher GPPS response rate might have relatively more patients visiting their practice more often instead of visiting an ED and therefore had more chance to experience long waiting times in the GP waiting room.

The increase of attendees at EDs started at the beginning of 2000s. This study only focused on the years 2009/10 to 2012/13. Studying earlier years and the most recent years might result in other findings. Furthermore, we were unable to include in this longitudinal study some of the factors tested in our cross-sectional study, such as percentage of patients able to book a convenient appointment to see a GP or a nurse, since comparable data over time were unavailable due to changes in questions in the GPPS [6], and factors unlikely to change over the period under study such as the geographical proximity to an ED and number of GP’s per population head or changes in national policy which would affect all the practices in the same way. We used the presented GPPS unweighted survey results as a result of a change in the GPPS weighting scheme from survey 2011/12 onwards resulting in incomparability of the data over time. By applying weights, GPPS aimed to generalize the findings to the whole general practice population by giving groups of patients with lower response more weight.^15^

GPPS response rate increased with age; patients over 65 responded more than three times than those 18–34, and more than twice than those aged 35–44, and women responded more often than men.^16^ Women older than 65 visit general practices twice as often as those aged 16–44, men older than 65 visit those practices at least three times more than those aged 16–44, [17] and also women visit those practices more often than men [18]. The response rate might then reflect the patients contacting the general practice. Therefore, using the unweighted GPPS data in an analysis and at the same time including the proportion of patients older than 65 and the percentage of female patients as co-variates might not only be a good alternative to using weighted data but might even better reflect the opinion of patients who most need primary health care.

By applying strict inclusion criteria we reduced bias in our data set and analyses, but a consequence is the lower proportion of general practices included in the financial year 2009/10 than in the other years. For a great part this is due to the fact that more general practices in that year had a very low reported self-referred discharged ED attendance rate. Those lower attendance rates might reflect recording or coding issues in that year and could be clustered around some hospitals or areas.

Our analysis of factors related to self-referred discharged ED attendance rate (our primary outcome) assumed that the likelihood of admission is equal for all EDs in England, conditional on these factors. However, if the same factors were positively related to the probability of admission for patients presenting themselves to EDs, this might result in our underestimating their impact on the primary outcome. Furthermore, associations of indicators changing over time with changes in emergency attendance strengthen evidence of causal relationships. However, given the ecological nature of the data (aggregated to general practice level), one cannot infer associations for individual patients.

### Comparison with existing literature

We tested the findings of a recent systematic review and a newly conducted cross-sectional study regarding several general practice factors that were identified to affect emergency care attendance [5, 6]. In our recent study we found no association between patients’ satisfaction with opening hours and emergency care attendance for the year 2012/13 [6]. To explain the absence of this relationship we previously suggested that general practices might have expanded their opening hours in the course of time. This study showed that although between-practice variation was not associated with emergency care attendance, combined emergency care attendance fell over time if more patients within a practice became satisfied with the general practice opening hours. Unlike Harris, Patel, and Bowen this study found an association between long waiting time at the practice and emergency attendance [13].

Evidence for continuity of care was mainly found in studies focusing on emergency care attendance abroad [5]. In our cross-sectional study we did not find an association between the proportion of patients having a preferred GP and ED attendance, and found an unexpected positive association between seeing the preferred GP often and ED attendance [6]. This longitudinal study shows that the proportion of patients having a preferred GP is more important than usually seeing the preferred GP when visiting the general practice; practices with a higher proportion of patients having a preferred GP were associated with lower ED attendance rates.

Findings on the effect of proportion of elderly patients are mixed as Cowling et al. found an inverse association, Baker et al. and Scantlebury et al. found no association, [9–11] and this study found a positive association with emergency attendance. However, we did not find a time specific effect. Several studies found a strong association between IMD and ED attendance [9–11, 13]. Following our cross-sectional study, [6] we included lower economic status measured by unemployment and practice populations’ health condition by life-expectancy instead of IMD. Higher unemployment and lower life-expectancy were associated with higher emergency care attendance rates, however, we did not find a time specific effect for unemployment.

A study using 40 English general practices and 20 A&E departments found a non-significant reduction in consultations for A&E departments and practices close to WiCs, while a study in Sheffield found a significant reduction in day-time ED attendance after the opening of a WiC [12, 19]. In the present study, local availability of WiCs or MIUs were associated with higher self-referred discharged ED attendance rates. As suggested in our cross-sectional study a likely explanation is that those alternative health care services were established by the NHS in areas with high ED attendance rates (or need for health care services) [6]. This longitudinal study found that activity at WiCs did not reduce ED attendance rates since the local availability of a WiC was associated with an increase in ED attendance that could not be compensated for in about 75% of the general practices having a WiC nearby. The increase in patients’ WiC attendance over time was not significantly associated with ED attendance. Furthermore, general practices that acquired a WiC did not see a reduction in their patients’ ED attendance rate. The activity at MIUs reduced ED attendance rates in about 40% of practices having a MIU nearby since a higher ED attendance rate associated with the local availability of a MIU could be compensated for in more general practices by their patients’ MIU attendance rates. In addition, in general practices where the MIU attendance rates increased over time, further reduction in their ED attendance rates was apparent. For MIU attendance greater reduction in self-referred discharged ED attendance rate was achieved than for WiC attendance, especially outside urban conurbations.

### Implications for general practices and directions for future research

Harris, Patel, and Bowen found that in north London about 70% of the self-referred ED attendances were repeated visits by the same individuals visiting ED twice or three times a year, and that a very small group of frequent attendees were responsible for 5% of all those attendances [13]. This study showed that general practices with a higher proportion of patients having a preferred GP were associated with lower ED attendance rates. Pinpointing general practices with higher number of frequent ED attendees and introducing or helping patients to be linked to a preferred GP might help to reduce ED attendance.

The association of general practice characteristics with ED attendance showed some relative small changes, i.e. a drop of 0.12 in the attendance rate with every percent increase of patients having a preferred GP. Following Cowling et al., [20] we might argue that the absolute effect is considerable given the huge number of patients attending at EDs. Furthermore, general practices with relatively more elderly, women or unemployed, or with lower patients’ life expectancy have a patient population that seems to have higher use of emergency services. This might reflect that these populations need more health care.

While self-referred ED attendance rates seem to stabilize over time, emergency care use including WiCs and MIUs still increases. This study showed some difference in associations of general practice and population characteristics with both emergency care usages. While satisfaction with practice opening hours was inversely associated with combined ED, WiC and MIU attendance, having a preferred GP was associated with self-referred discharged ED attendance only. Furthermore, the proportion of UK-white patients was positively associated only with the combined ED, WiC and MIU attendance use. This might indicate that practice populations with a higher proportion of UK-white patients were more aware of alternative health care services such as MIU and WiCs or those services were better available to them.

Further investigations should consider a wider span of years, including data from 2013 onwards to monitor potential effects of any changes in practice policies on access and continuity. Longitudinal analysis allows the evaluation of variables which both vary between and within general practices. Previous analyses including the present study have analyzed data aggregated to the practice level, but data at an individual level could be particularly informative concerning the influence of practice determined characteristics which may apply differently to individual patients, for example, continuity of care. Such studies have been done abroad, but have not been conducted in the UK as far as we know [21–23]. We have established the most likely primary care factors that are linked to the increasing demand on emergency departments and this should inform policy on primary care delivery for a sustainable acute care pathway.

## Conclusions

This study showed that improvements in general practice access and continuity of primary care could reduce ED attendance rate. As more general practices in the UK are merging into larger ‘super-practices’, they introduce new forms of access and as a result improve accessibility [24]. The introduction in 2014 of an UK government scheme ‘named GP’ for each patient aged 75 and over who is responsible for their health care is aimed to improve health outcomes and to reduce hospitalisation by increasing continuity of care. Such an introduction might especially be profitable for practices with relatively more elderly patients as these practices seem to have higher emergency service attendance rates. Practices having relatively more female or unemployed patients among their practice population also showed higher emergency service attendance rates reflecting that these groups also need more health care. To reduce the ED attendance rates among those practices additional support or measures might be needed such as the extension of the ‘named GP’ scheme to all patients in 2015 [25]. Furthermore, an increase in the use of MIUs might also decrease ED attendance rate. As both MIUs and WiCs can be seen either as substitutes for ED use or as complementary primary care services, our results suggest that MIUs could act as substitutes for EDs. Establishing an MIU nearby a hospital with an ED might support or improve the use of MIUs.

## Additional file

Additional file 1:
**Figure S1.** Net reduction in self-referred discharged ED attendance rate in relation to Minor Injuries Unit (MIU) or Walk-in Centre (WiC) attendance rates (see **Table S1.** for values of centiles). **Figure S2.** Local availability of alternative health care and self-referred discharged ED attendance rates. **Box 1.** Accident and Emergency (A&E) department types in England. **Table S1.** Time average and time specific rates per 1,000 ED attendances of centiles in **Figure S1.** **Table S2.** Estimates of B-coefficients from multilevel regression models for the association between general practice characteristics and socio-demographic profile of patients and ED attendance in England, 2009/10-2012/13. **Table S3.** Estimates of B-coefficients from multilevel regression models for the association between general practice characteristics and socio-demographic profile of patients and combined ED, MIU & WiC attendance in England, 2009/10-2012/13. (DOCX 102 kb)

## Abbreviations

A&E: Accident and Emergency
ED: Emergency Departments
GPPS: GP Patient Survey
IMD: Index of multiple deprivation
MIU: Minor Injuries Unit
PCT: Primary Care Trusts
WiC: Walk-in Centre

## Footnotes

1
https://www.gov.uk/government/statistics/accident-and-emergency-weekly-data-week-ending-13-july-2014 (accessed 5th February 2015.

2 In the UK patients are registered at one family practice and usually attend that practice.

3
https://www.nhscomparators.nhs.uk/NHSComparators/ (accessed 26th–27th January 2015); was a free website until 31st March 2015.

4 GP Patient Survey 2009–10 to 2012–13 are available at https://gp-patient.co.uk/surveys-and-reports
 (accessed between 9th and 24th April 2015)

5 See the surveys and the technical reports for each year at https://gp-patient.co.uk/surveys-and-reports (accessed 2nd February 2015)

6
https://gp-patient.co.uk/default?pageid=33 (accessed 13th March 2015)

7 See note 3.

8 Office National Statistics available at https://geoportal.statistics.gov.uk/ (accessed 18th August 2014).

9 See note 3.

10
https://www.gov.uk/government/collections/english-indices-of-deprivation (accessed 16th January 2015).

11 National General Practice Profiles male and female life expectancy 2006–2010 available at http://fingertips.phe.org.uk/profile/general-practice (accessed 7th August 2014).

12 See note 3.

13 See note 2.

14 PCTs work with Local Authorities and other agencies that provide health and social care locally to make sure that local community’s needs are being met (http://www.datadictionary.nhs.uk/). By including them as a third level we take into account clustering.

15 See note 5.

16 See note 5.
