# Supplementary material for: Exploring the relationship between general practice characteristics and attendance at Walk-in Centres, Minor Injuries Units and Emergency Departments in England 2009/10–2012/2013: a longitudinal study
Source: BMC Health Serv Res. 2017 Aug 8;17:546. doi: 10.1186/s12913-017-2483-x (PMC5549356; doi:10.1186/s12913-017-2483-x)
Supplement: Additional file 1: — Figure S1. Net reduction in self-referred discharged ED attendance rate in relation to Minor Injuries Unit (MIU) or Walk-in Centre (WiC) attendance rates (see Table S1. for values of centiles). Figure S2. Local availability of alternative health care and self-referred discharged ED attendance rates. Box 1. Accident and Emergency (A&E) department types in England. Table S1. Time average and time specific rates per 1,000 ED attendances of centiles in Figure S1. Table S2. Estimates of B-coefficients from multilevel regression models for the association between general practice characteristics and socio-demographic profile of patients and ED attendance in England, 2009/10-2012/13. Table S3. Estimates of B-coefficients from multilevel regression models for the association between general practice characteristics and socio-demographic profile of patients and combined ED, MIU & WiC attendance in England, 2009/10-2012/13. (DOCX 102 kb) [file 12913_2017_2483_MOESM1_ESM.docx]

Supplementary Figure 1: Net reduction in self-referred discharged ED attendance rate in relation to Minor Injuries Unit (MIU) or Walk-in Centre (WiC) attendance rates (see supplementary Table 1 for values of centiles).


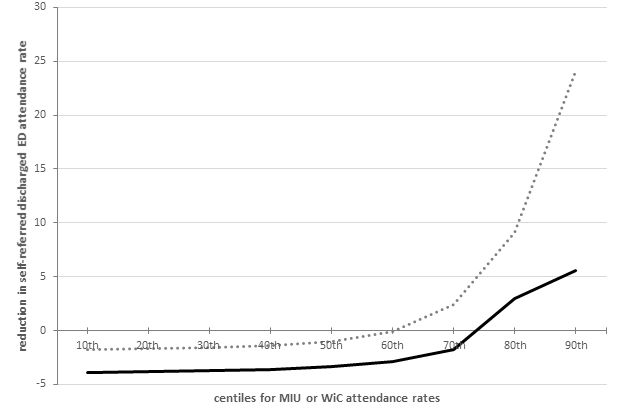


WiC MIU

Supplementary Figure 2: Local availability of alternative health care and self-referred discharged ED attendance rates.


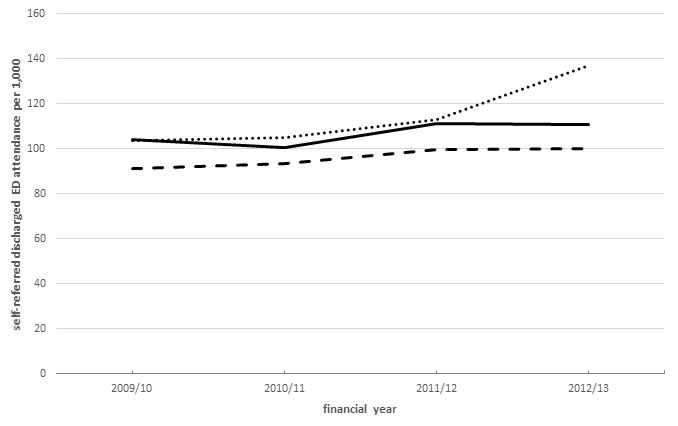


never Walk-in Centre (WiC) and Minor Injuries Unit (MIU) around

only WiC after 2009/10

only MIU after 2009/10

Supplementary Box 1: Accident and Emergency (A&E) department types in England

| A&E department types following the definitions used in the English National Health Service (NHS) data dictionary.    **Type 1 A&E department (Major A&E)**  Emergency departments are a consultant led 24 hour service with full resuscitation facilities and designated accommodation for the reception of accident and emergency patients.  **Type 2 A&E department (Single Specialty)**  Consultant led mono specialty accident and emergency service (e.g. ophthalmology, dental) with designated accommodation for the reception of patients.  **Type 3 A&E department (Other A&E / Minor Injury Unit)**  Other type of A&E/minor injury activity with designated accommodation for the reception of accident and emergency patients. The department may be doctor led or nurse led and treats at least minor injuries and illnesses and can be routinely accessed without appointment. Excludes NHS walk-in centres.  **Type 4 A&E department**  NHS walk-in centres |
| --- |

Supplementary Table S1: Time average and time specific rates per 1,000 ED attendances of centiles in supplementary Figure 1.

| **Centiles** | **Walk-in Centres** | | **Minor Injuries Units** | |
| --- | --- | --- | --- | --- |
|  | *Time average* | *Time specific* | *Time average* | *Time specific* |
| 10th | 0.5 | 1.2 | 1.1 | 1.3 |
| 20th | 0.8 | 1.6 | 1.5 | 1.6 |
| 30th | 1.1 | 2.2 | 2.1 | 2.1 |
| 40th | 1.7 | 3.2 | 3.0 | 2.7 |
| 50th | 2.7 | 4.8 | 5.1 | 3.9 |
| 60th | 5.0 | 7.9 | 10.0 | 6.7 |
| 70th | 9.3 | 15.7 | 23.4 | 14.9 |
| 80th | 17 | 30.4 | 44.8 | 40.7 |
| 90th | 39.9 | 73.4 | 83.35 | 101.9 |

Supplementary Table 2. Estimates of B-coefficients from multilevel regression models for the association between general practice characteristics and socio-demographic profile of patients and ED attendance in England, 2009/10-2012/13.

|  | **Model S1: time average predictors only** | | **Model S2: including time specific predictors in addition to time average predictors** | | **Model S3: including GPPS response rate, and local availability of MIU and WiC, in addition to predictors in model S2** | |
| --- | --- | --- | --- | --- | --- | --- |
| **Fixed part** | b-coef. | 95% CI | b-coef. | 95% CI | b-coef. | 95% CI |
| Intercept | 430.698*** |  | 430.279*** |  | 399.741*** |  |
| 2010-11 ref. 2009-10 | 9.624*** | 8.691,10.557 | 9.965*** | 9.025,10.905 | 8.920*** | 7.949,9.890 |
| 2011-12 ref. 2009-10 | 16.070*** | 15.132,17.008 | 18.614*** | 17.462,19.766 | 17.124*** | 15.902,18.346 |
| 2012-13 ref. 2009-10 | 16.215*** | 15.283,17.146 | 19.801*** | 18.445,21.157 | 17.343*** | 15.885,18.801 |
| Time specific pct. 65+ |  |  | -0.041 | -0.173,0.092 | -0.125~ | -0.259,0.009 |
| Time average pct. 65+ | 0.627*** | 0.513,0.742 | 0.670*** | 0.495,0.845 | 0.851*** | 0.669,1.032 |
| Time specific pct. female patients |  |  | -0.058 | -0.182,0.066 | -0.08 | -0.203,0.044 |
| Time average pct. female patients | 0.319** | 0.079,0.559 | 0.395** | 0.126,0.665 | 0.441** | 0.172,0.710 |
| Time specific pct. UK-white patients |  |  | -0.07 | -0.205,0.065 | -0.063 | -0.198,0.072 |
| Time average pct. UK-white patients | -0.067~ | -0.141,0.006 | 0.002 | -0.152,0.155 | 0.036 | -0.118,0.190 |
| Time specific pct. unemployed patients |  |  | 0.001 | -0.222,0.223 | -0.003 | -0.225,0.219 |
| Time average pct. unemployed patients | 2.255*** | 1.880,2.630 | 2.239*** | 1.807,2.672 | 2.068*** | 1.634,2.503 |
| Time specific pct. satisfied open hours |  |  | -0.101~ | -0.213,0.011 | -0.089 | -0.201,0.022 |
| Time average pct. satisfied open hours | -0.086 | -0.214,0.043 | 0.014 | -0.157,0.184 | 0.048 | -0.122,0.218 |
| Time specific pct. waited too long |  |  | 0.105 | -0.055,0.265 | 0.099 | -0.061,0.259 |
| Time average pct. waited too long | 0.326*** | 0.191,0.460 | 0.218* | 0.008,0.427 | 0.201~ | -0.008,0.410 |
| Time specific pct. had pref. GP |  |  | 0.01 | -0.068,0.088 | 0.014 | -0.064,0.092 |
| Time average pct. had pref. GP | -0.103*** | -0.153,-0.054 | -0.113* | -0.206,-0.021 | -0.075 | -0.168,0.018 |
| Time specific pct. often spoke to pref. GP |  |  | -0.047 | -0.110,0.017 | -0.043 | -0.106,0.021 |
| Time average pct. often spoke to pref. GP | -0.011 | -0.063,0.041 | 0.036 | -0.046,0.118 | 0.016 | -0.067,0.098 |
| Time specific WiC attendance rate |  |  | 0.004 | -0.013,0.020 | -0.011 | -0.029,0.006 |
| Time average WiC attendance rate | -0.221*** | -0.278,-0.164 | -0.226*** | -0.286,-0.167 | -0.226*** | -0.285,-0.167 |
| Time specific MIU attendance rate |  |  | -0.205*** | -0.216,-0.193 | -0.205*** | -0.216,-0.194 |
| Time average MIU attendance rate | -0.275*** | -0.293,-0.257 | -0.061*** | -0.083,-0.040 | -0.062*** | -0.083,-0.040 |
| Cities and towns ref. urban conurbation | 4.037** | 1.193,6.881 | 3.908** | 1.063,6.753 | 4.457** | 1.620,7.294 |
| Rural areas ref. urban conurbation | -8.454*** | -11.604,-5.303 | -8.477*** | -11.628,-5.325 | -6.852*** | -10.020,-3.684 |
| Male life expectancy 2006/10 | -4.795*** | -5.226,-4.363 | -4.823*** | -5.254,-4.392 | -4.399*** | -4.846,-3.952 |
| local availability of MIU |  |  |  |  | 2.097*** | 1.071,3.123 |
| local availability of WiC |  |  |  |  | 4.010*** | 2.504,5.517 |
| GPPS response rate |  |  |  |  | -0.395*** | -0.503,-0.287 |
| **Random components of variance** |  |  |  |  |  |  |
| *PCT level:*  Intercept | 864.388*** | 666.374,1062.402 | 866.410*** | 667.584,1065.237 | 861.930*** | 664.492,1059.369 |
| *General practice level:* Intercept | 451.636*** | 430.321,472.950 | 463.993*** | 442.721,485.265 | 459.457*** | 438.344,480.571 |
| *Year level:* Intercept | 674.316*** | 660.750,687.882 | 631.182*** | 618.482,643.882 | 629.819*** | 617.146,642.492 |
| **Statistics** |  |  |  |  |  |  |
| N | 26498 |  | 26498 |  | 26498 |  |
| deviance | 257446.81 |  | 256189.77 |  | 256091.69 |  |
| Log likelihood | -128723.41 |  | -128094.88 |  | -128045.84 |  |

*** p<0.001, ** p<0.01, * p<0.05, ~ p<0.1

Supplementary Table 3. Estimates of B-coefficients from multilevel regression models for the association between general practice characteristics and socio-demographic profile of patients and combined ED, MIU & WiC attendance in England, 2009/10-2012/13.

|  | **Model S1: time average predictors only** | | **Model S2: including GPPS response rate in addition to time average and time specific indicators** | |
| --- | --- | --- | --- | --- |
| **Fixed part** | b-coef. | 95% CI | b-coef. | 95% CI |
| Intercept | 461.017*** |  | 421.933*** |  |
| 2010-11 ref. 2009-10 | 12.279*** | 10.809,13.748 | 11.106*** | 9.550,12.662 |
| 2011-12 ref. 2009-10 | 33.081*** | 31.604,34.558 | 33.472*** | 31.620,35.325 |
| 2012-13 ref. 2009-10 | 43.380*** | 41.913,44.847 | 42.711*** | 40.518,44.905 |
| Time specific pct. 65+ |  |  | -0.129 | -0.347,0.089 |
| Time average pct. 65+ | 1.042*** | 0.879,1.206 | 1.285*** | 1.005,1.565 |
| Time specific pct. female patients |  |  | -0.11 | -0.312,0.091 |
| Time average pct. female patients | 0.449* | 0.106,0.793 | 0.589** | 0.192,0.986 |
| Time specific pct. UK-white patients |  |  | -0.338** | -0.557,-0.119 |
| Time average pct. UK-white patients | 0.029 | -0.076,0.135 | 0.419*** | 0.176,0.663 |
| Time specific pct. unemployed patients |  |  | -0.085 | -0.446,0.276 |
| Time average pct. unemployed patients | 3.065*** | 2.526,3.604 | 2.914*** | 2.265,3.564 |
| Time specific pct. satisfied open hours |  |  | -0.260** | -0.441,-0.078 |
| Time average pct. satisfied open hours | -0.155~ | -0.339,0.028 | 0.158 | -0.101,0.416 |
| Time specific pct. waited too long |  |  | -0.033 | -0.293,0.226 |
| Time average pct. waited too long | 0.506*** | 0.314,0.698 | 0.514** | 0.190,0.837 |
| Time specific pct. had pref. GP |  |  | 0.05 | -0.076,0.177 |
| Time average pct. had pref. GP | -0.063 | -0.134,0.008 | -0.062 | -0.208,0.084 |
| Time specific pct. often spoke to pref. GP |  |  | 0.027 | -0.076,0.130 |
| Time average pct. often spoke to pref. GP | -0.008 | -0.082,0.067 | -0.054 | -0.182,0.074 |
| Cities and towns ref. urban conurbation | -5.356** | -9.368,-1.345 | -4.843* | -8.850,-0.836 |
| Rural areas ref. urban conurbation | -18.299*** | -22.751,-13.847 | -16.329*** | -20.821,-11.837 |
| Male life expectancy 2006/10 | -5.442*** | -6.058,-4.826 | -4.882*** | -5.525,-4.238 |
| GPPS response rate |  |  | -0.486*** | -0.650,-0.322 |
| **Random components of variance** |  |  |  |  |
| *PCT level:* Intercept | 1589.722*** | 1224.157,1955.286 | 1592.432*** | 1226.289,1958.576 |
| *General practice level:* Intercept | 833.743*** | 790.005,877.481 | 828.632*** | 785.077,872.187 |
| *Year level*: Intercept | 1676.937*** | 1643.222,1710.652 | 1675.020*** | 1641.344,1708.697 |
| **Statistics** |  |  |  |  |
| N | 26498 |  | 26498 |  |
| deviance | 280084.63 |  | 280031.5 |  |
| Log likelihood | -140042.31 |  | -140015.75 |  |

*** p<0.001, ** p<0.01, * p<0.05, ~ p<0.1
